# Deciphering Structural Photophysics of Fluorescent Proteins by Kinetic Crystallography

**DOI:** 10.3390/ijms18061187

**Published:** 2017-06-02

**Authors:** Dominique Bourgeois

**Affiliations:** Institut de Biologie Structurale, Univ. Grenoble Alpes, CNRS, CEA, CNRS, IBS, F-38000 Grenoble, France; dominique.bourgeois@ibs.fr; Tel.: +33-4-57-42-86-44

**Keywords:** fluorescent proteins, phototransformations, protein dynamics, in crystallo optical spectroscopy, kinetic X-ray crystallography, quantum mechanics/molecular mechanics, single molecule imaging

## Abstract

Because they enable labeling of biological samples in a genetically-encoded manner, Fluorescent Proteins (FPs) have revolutionized life sciences. Photo-transformable fluorescent proteins (PTFPs), in particular, recently attracted wide interest, as their fluorescence state can be actively modulated by light, a property central to the emergence of super-resolution microscopy. PTFPs, however, exhibit highly complex photophysical behaviours that are still poorly understood, hampering the rational engineering of variants with improved performances. We show that kinetic crystallography combined with in crystallo optical spectroscopy, modeling approaches and single-molecule measurements constitutes a powerful tool to decipher processes such as photoactivation, photoconversion, photoswitching, photoblinking and photobleaching. Besides potential applications for the design of enhanced PTFPs, these investigations provide fundamental insight into photoactivated protein dynamics.

## 1. Introduction

### 1.1. Fluorescent Proteins

Fluorescence microscopy is a key technique for cell biologists, relying heavily on the use of fluorescent proteins (FPs) as markers [1]. FPs form 11-stranded β-barrels that encompass a three-residue-based 4-(para-hydroxybenzylidene)-5-imidazolinone (p-HBI) endogenous chromophore. Chromophore maturation requires only oxygen as an external cofactor, so that, under aerobic conditions, fluorescence can be considered genetically encoded. The high fluorescence quantum yield of FPs is due to the fact that the chromophore is held rigidly within the protein matrix through tight non-covalent interactions, preventing non-radiative relaxation pathways upon light excitation. Indeed, free p-HBI in solution does not fluoresce. However, fluorescence of FPs is typically highly unstable, in line with the notion of a dynamical chromophore breathing within a complex conformational landscape [2].

The dynamic properties of fluorescent proteins (for reviews, see [3,4,5,6,7]) have been subjected to a huge number of photophysical investigations by various spectroscopies including ultrafast, fluorescence correlation, infrared, resonant-Raman and single-molecule approaches. In parallel, the crystallographic structures of a large number of FPs have been determined (~330 protein databank (PDB) entries), presenting a detailed but most often static molecular view. Despite insightful structure-based simulations of light-induced dynamical behaviour, it has generally remained a challenge to precisely link the structure, spectroscopy and dynamics of FPs.

A large number of FPs have been engineered from *Aequora victoria* GFP or from various Anthozoan marine organisms such as corals or anemones, providing fluorescent colours across the entire optical spectrum (450 to 650 nm) [8]. In parallel, a new set of FPs termed phototransformable FPs (PTFPs) have become the focus of intensive research due to many potential applications in advanced microscopy and biotechnology [5,9]. The fluorescent state (on/off) or emission colour (e.g., green/red) of PTFPs can be precisely controlled by light, forming the core principle of several super resolution microscopy techniques such as PALM (“photo activated localization microscopy”) or RESOLFT (“reversible saturable optical fluorescence transitions”) [10]. In this context, mechanistic investigations of PTFPs have recently become the subject of focused investigation so as to rationally design variants with enhanced photophysical properties [7,11,12,13,14,15,16,17]. This requires correlating structural and spectroscopic information with a dynamical perspective.

One way to achieve this goal consists of using kinetic crystallography, i.e., inducing conformational changes in crystallo by reaction triggering and solving the structures of the resulting transient states [18,19]. By combining kinetic crystallography with in crystallo optical spectroscopy, the subtle structural changes associated with specific photophysical states can be directly monitored. These experimental data may then be used as input for further modelling, for example based on quantum mechanics/molecular mechanics (QM/MM) methods, to provide refined mechanisms.

How such a combination of approaches may improve our understanding of PTFP photophysics and provide general insight into photoactivated protein dynamics in these fascinating markers is the focus of this chapter. Based on the example of IrisFP, a single mutant of EosFP that exhibits multiple phototransformations [20] and crystallizes particularly well, we review how mechanisms such as photoconversion, photoswitching, and photoblinking; photobleaching is associated with specific structural changes, highlighting the subtle conformational plasticity of these supposedly “rigid” macromolecules.

### 1.2. Various Phototransformation in Photo-Transformable Fluorescent Proteins

**Figure 1:**
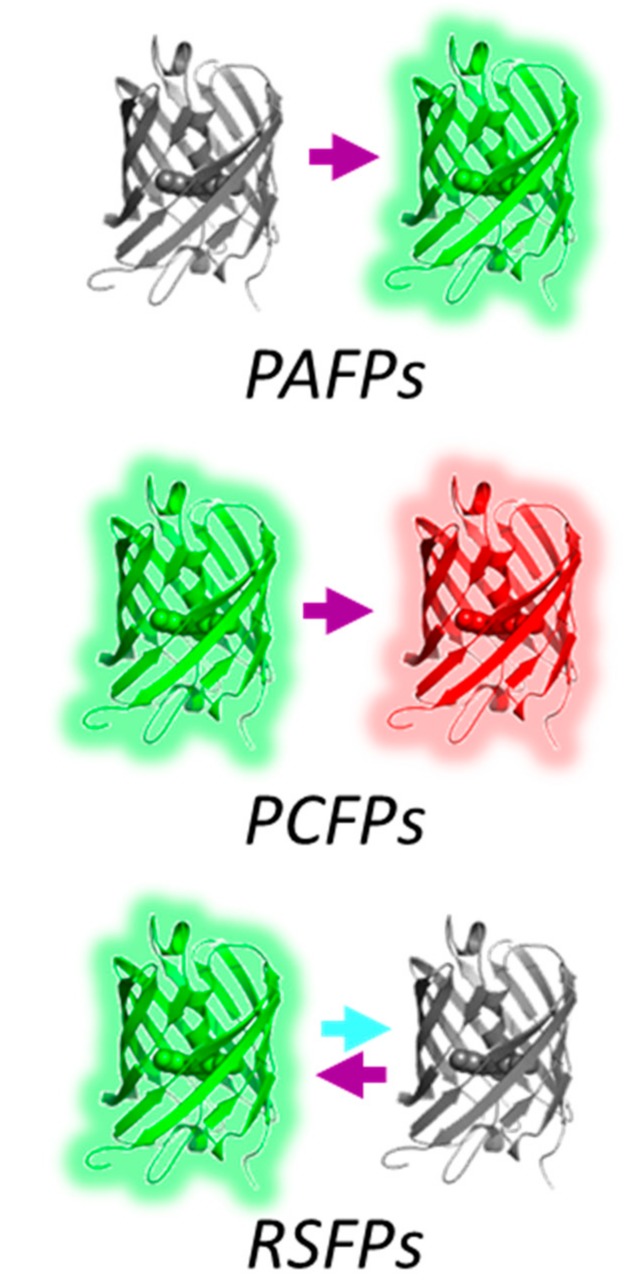
Main phototransformation pathways in PTFPs. Arrows are color-coded according to the wavelengths that generate the phototransformations.

Three types of phototransformations may be distinguished in PTFPs (Figure 1): non-reversible activation from a nonfluorescent to a fluorescent state (“photoactivation”), non-reversible conversion between two fluorescent states with different emission wavelengths (“photoconversion”); reversible switch between a fluorescent on-state and a non-fluorescent off-state (“photoswitching”). In addition, like all fluorophores, PTFPs undergo transient stochastic switching events to non-fluorescent dark states (“blinking”); eventual conversion to a permanent off state (“bleaching”). It is essential to note that phototransformations, blinking and bleaching are typically low quantum yield processes (q ≈ 10^−2^–10^−5^). This makes the real-time observation of a particular pathway at the ensemble level very challenging because it is practically impossible to direct all molecules in a sample down this pathway. What one can expect to see are precursor states common to several pathways or a complex superposition of different states. However, phototransformation product species are typically thermally stable for at least minutes, so, contrary to intermediate states, their end-structure can be relatively easily captured by simple freeze trapping.

## 2. Principles of Kinetic Crystallography

**Figure 2:**
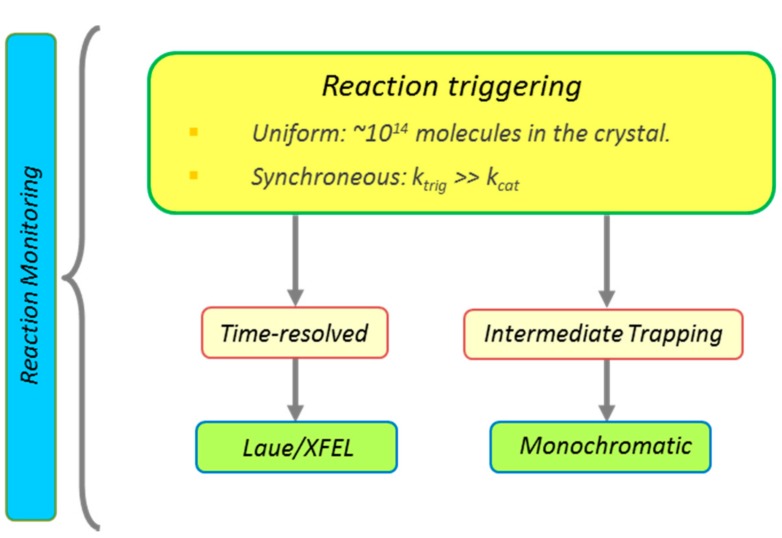
Principles of kinetic crystallography.

The main principles of kinetic crystallography are reported in Figure 2 [18,19]. Two main strategies may be followed: real-time or trapping. Real-time approaches are in principle very powerful as they provide both kinetic and structural data on the ultrafast timescale. They typically rely on pump-probe data collection schemes based either on Laue crystallography at synchrotron sources (achievable time resolution: ~100 picoseconds) [21,22], or on serial crystallography at X-ray free electron lasers (XFEL) sources (achievable time resolution: ~100 femtoseconds) [23]. Due to issues related to X-ray induced radiation damage and poor penetration of actinic light into the bulk of thick crystals, the Laue approach has so far not been successfully used to study fluorescent proteins. These difficulties are alleviated when microcrystals are used at XFEL sources, which presently open new opportunities for the detection of short-lived intermediate species [24], possibly in electronically excited states. However, the extreme laser power density required to excite a sufficient fraction of molecules within each crystal (typically > GW/cm^2^), together with the multiple phototransformation pathways in PTFPs, are expected to present considerable challenges for the precise assignment of the exact nature of the possibly detected transient states. Preliminary ultrafast spectroscopic investigations, preferably in crystallo, are required for prediction of the most interesting time points to be collected and to provide control experiments, e.g., to assess the consequences of using large laser excitation powers [25].

Temperature-based trapping approaches are easier to set up but suffer from two main weaknesses: low temperatures may alter the conformational landscape of macromolecules and kinetic rates cannot be extracted easily from such experiments [19]. Nevertheless, most insight into fluorescent protein structural photophysics has so far been derived from such approaches. Depending on the genuine lifetime of the intermediate of interest, two trapping strategies may be used. Long-lived intermediate states (>~1 s) may be trapped by the “trigger freeze” approach, in which light-induced reaction initiation is performed at room temperature and the formed intermediate is then captured by flash cooling after an appropriate delay. Short-lived intermediate states (<~1 s) cannot be trapped in this way because flash cooling typically takes hundreds of milliseconds, so that the “freeze trigger” approach should rather be used. Here, the crystal is first cooled to cryogenic temperature so that the reaction cannot proceed to the product state—only then is illumination applied. An intermediate state protected by a free energy barrier that cannot be passed at the used temperature may then accumulate under steady-state illumination. Similarly to real-time-resolved experiments, complementary monitoring by optical spectroscopy in crystallo is essential to characterize the nature of the trapped states. Dedicated microspectrophotometers have been constructed for this purpose [26].

Three important considerations should be kept in mind when conducting kinetic crystallography experiments: (i) the rate of reaction triggering should always be faster than the rate of disappearance of the intermediate of interest, except in conditions of steady-state accumulation of the intermediate; (ii) reaction triggering should be as homogeneous as possible throughout the crystal volume, so that the occupancy of the intermediate state is sufficient for unambiguous detection. The usually very high optical density of light-sensitive crystals may seriously impact the achievable triggering homogeneity; and (iii) the crystalline state may considerably alter reaction rates and can significantly restrict the amplitudes of the genuine structural changes. Local, small-scale rearrangements are thus more easily captured in kinetic crystallography experiments than large rearrangements.

Finally, when dealing with chromophore embedding macromolecules such as fluorescent proteins, X-ray induced photochemistry should always be considered. Indeed, when impinging on solvent or protein molecules, X-rays release a large quantity of free electrons that can be viewed as a strong reduction potential and can easily react with electron-affine moieties such as conjugated systems [27].

It is important to note that, under some circumstances, the two main kinetic crystallography approaches described above can be combined, i.e., time-resolved experiments can be conducted at cryogenic temperatures. This would, for example, be the case if energy barriers between several intermediates are low enough so that interconversion rates between those intermediates remain significant at the cryo temperature used [28].

## 3. IrisFP: A Well-Suited Candidate for Kinetic Crystallography of PTFPs

**Figure 3:**
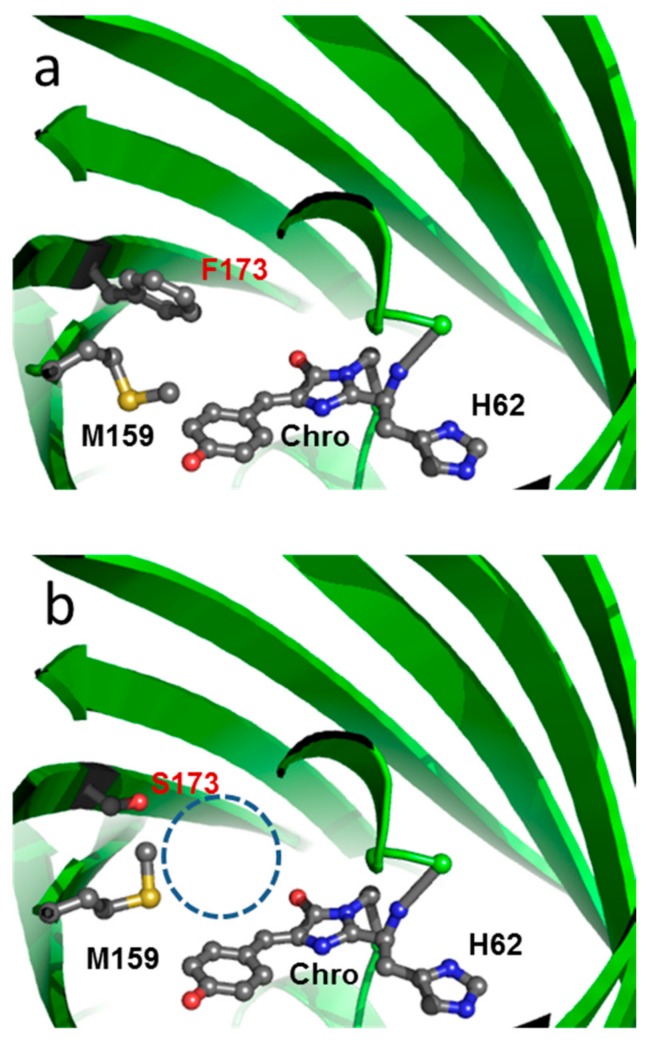

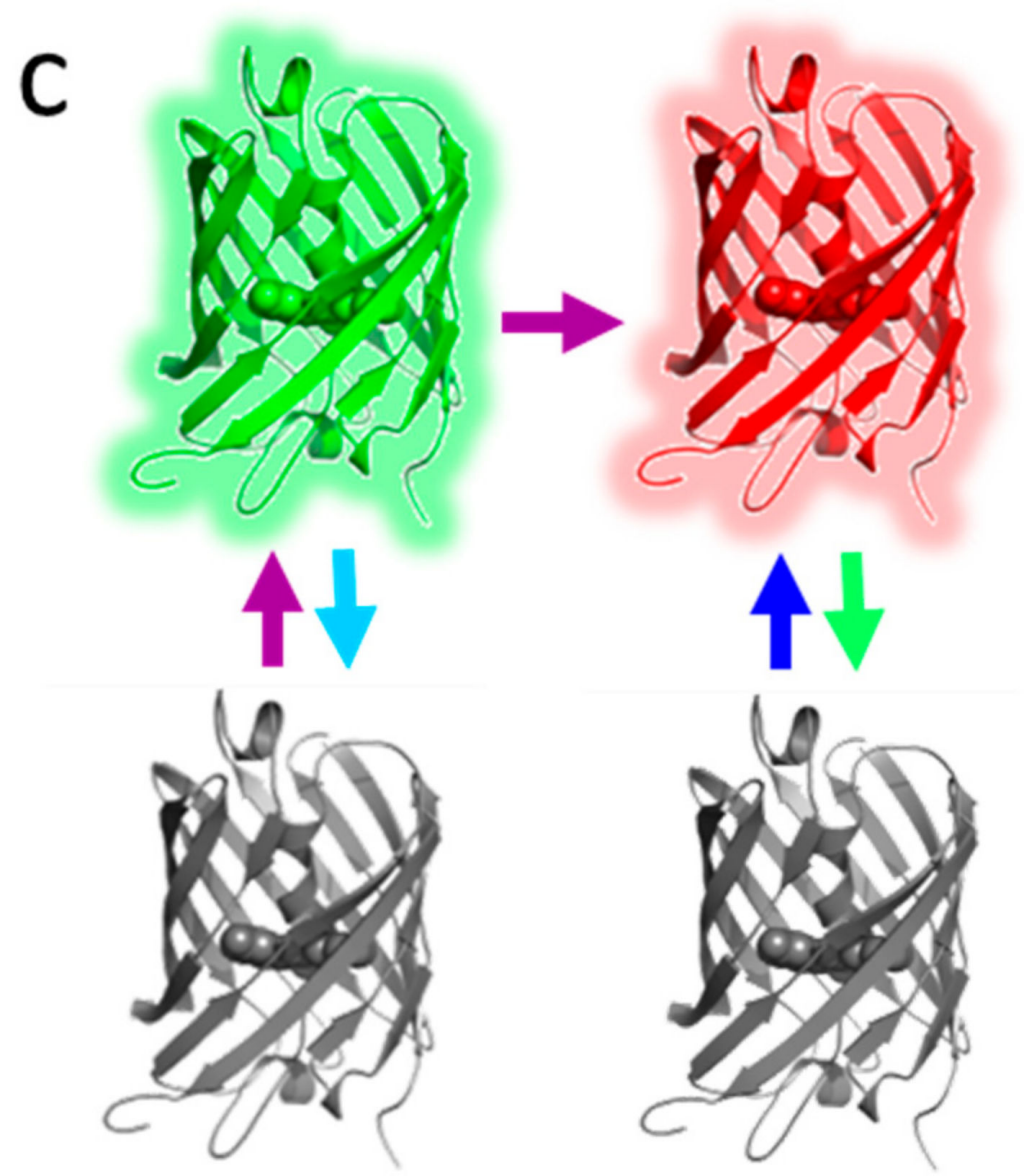
Multiple phototransformations of IrisFP. (**a**) Structure of the chromophore pocket of EosFP, the IrisFP parent. The histidine at position 62 is characteristic of green to red photoconvertible FPs. EosFP has a phenylalanine at position 173; (**b**) Structure of the chromophore pocket of IrisFP. The single mutation F173S creates empty space above the chromophore (dashed circle) and confers its photoswitching properties to IrisFP; (**c**) View of the multiple phototransformations in IrisFP. Arrows are color-coded according to the wavelengths that generate photoconversion or photoswitching.

IrisFP was discovered upon serendipitous mutagenesis of EosFP, a green-to-red photoconvertible fluorescent protein (PCFP) from the stony coral *Lobophyllia hemprichii* [20]. The central feature of Anthozoan PCFPs is that they possess a histidine as the first amino acid of the chromophore. The mutation F173S, by way of increasing the size of the chromophore pocket (Figure 3a), confers additional photoswitching properties to IrisFP (EosFP-F173S). In total, IrisFP can be reversibly switched on and off in both its green and red states (Figure 3b). This PTFP is also tetrameric, which, although not a desirable property for imaging applications, facilitates robust crystallization. Crystals of IrisFP are rod-shaped, reaching typical sizes of 100 × 100 × 500 µm^3^. Such crystal morphology is well suited to kinetic crystallography experiments. Indeed, diffraction data sets from both an illuminated and a non-illuminated part of the sample can be collected on the same crystal, allowing the calculation of experimental difference electron density maps essentially free of systematic errors [29,30]. Such maps are able to highlight subtle light-induced structural changes occurring on a relatively small fraction of the molecules much more clearly than standard difference electron density maps between an experimental and a calculated set of structure factor amplitudes. However, this is only true if no significant light-induced cell expansion occurs in the illuminated part of the crystal, so care has to be taken not to over illuminate the samples, even if this is at the cost of a reduced occupancy of the photophysical state of interest. The rod-shaped IrisFP crystals, if they are not too thick, are also convenient for the recording of optical spectra that are essential to the correct interpretation of crystallographic data. Even if the tetrameric nature of IrisFP crystals makes the crystallographer’s life easier, it should not be forgotten that the oligomeric status of PTFPs may impact their photophysical behaviour [16], so that findings made for IrisFP should not be extrapolated to monomeric variants such as mIrisFP [31] without care.

## 4. Green to Red Photoconversion

**Figure 4:**
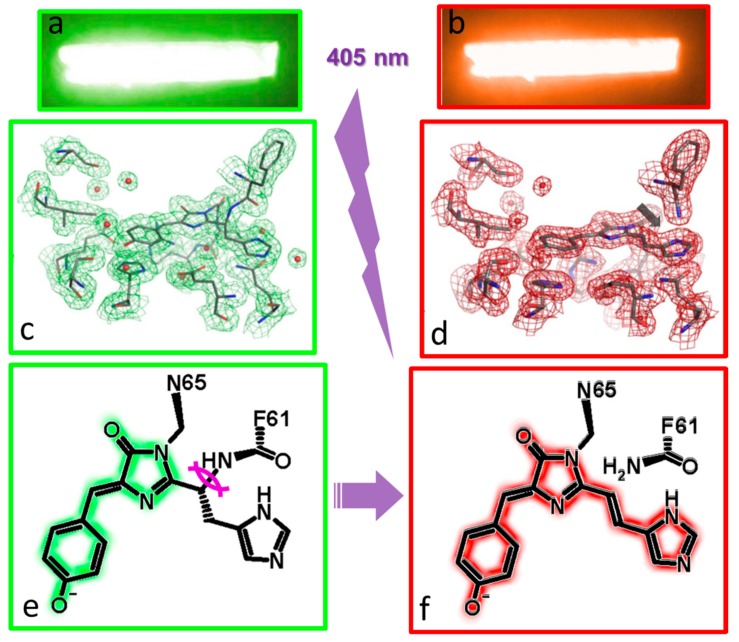
Green to red photoconversion of IrisFP. (**a**,**b**) fluorescence emission by an IrisFP crystal in the green and red states; (**c**,**d**) electron density maps of the chromophore pocket of IrisFP in the green and red states, overlaid on refined models of the protein in the two states. The backbone breakage between Phe61 and His62 is clearly visible; (**e**,**f**) scheme of the chemical reaction: the UV induced (purple lightning) backbone breakage (curved violet line) results in an elongation of the conjugated electron system of the chromophore, producing the red shift.

Green-emitting native IrisFP crystals appear yellow under brightfield illumination essentially due to a main absorbance band centered at around 490 nm characteristic of the chromophore in its anionic state. Upon gentle illumination with violet (405 nm) laser light, crystals left in their crystallization tray progressively turn red, revealing in crystallo photoconversion (Figure 4). As compared to the green-state structure obtained from a non-illuminated crystal, the red-state structure of IrisFP reveals cleavage of the peptide bond between the amide nitrogen and the α-carbon of His62, the first amino acid of the chromophore. Similar structural changes associated with green-to-red photoconversion have been observed in all PCFPs investigated by crystallography thus far, including Dendra2 [32], Kaede [33], KikGR [34], pcDronpa [16] and a least-evolved ancestral PCFP [35]. A double bond on the His62 side chain is formed, leading to extension of the chromophore conjugated π-system and the associated red shift. Apart from these changes and the disappearance of a water molecule flanking His62, the three-dimensional structures of IrisFP remain very similar in the green and red forms. Interestingly, to date, no intermediate along the photoconversion pathway has been detected experimentally, neither spectroscopically nor crystallographically, most likely due to the very small quantum yield of the process (~10^−4^). However, substantial free energy barriers must be crossed along the reaction, as photoconversion does not proceed at all at cryogenic temperature. Putative mechanisms such as those based on excited state proton transfer (ESPT) and a β-elimination reaction have been proposed following photochemical consideration and modeling studies [36,37]. A recent intriguing proposal involves twisting of the chromophore as a pre-requisite for disrupting the charge network and activating proton shuttling [35]. Further investigations, including at the single-molecule level, are needed to pinpoint the nature of intermediates along the photoconversion pathway.

## 5. On-Off Photoswitching

**Figure 5:**
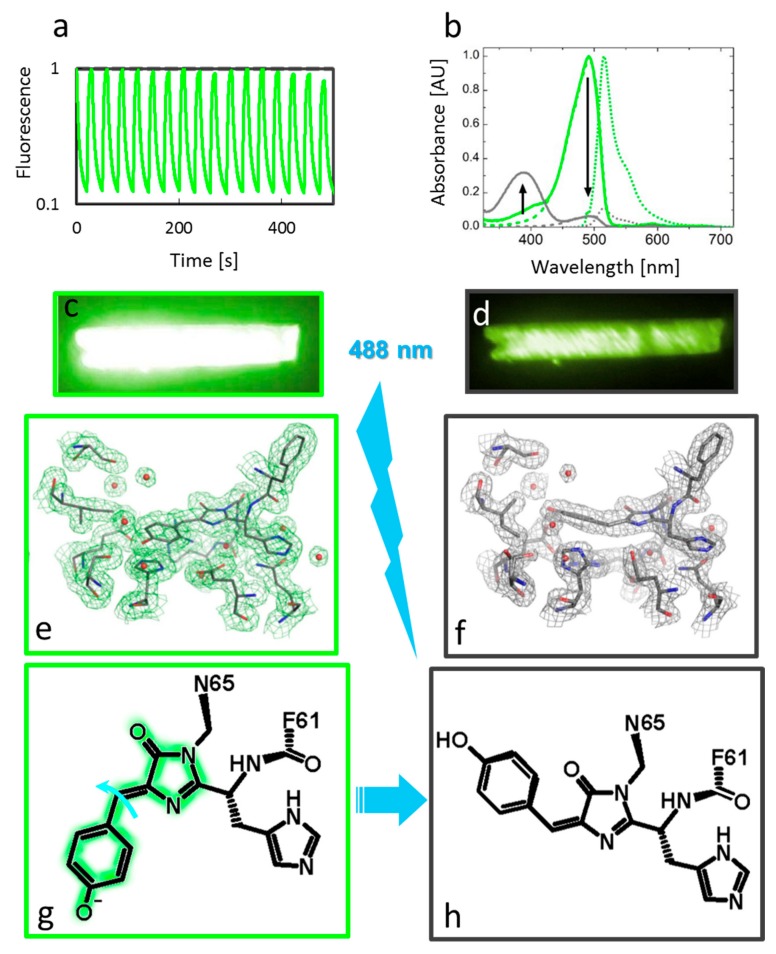
On to off photoswitching of IrisFP. (**a**) Fluorescence switching in crystalline IrisFP stimulated by alternate 488 and 405 nm illumination; (**b**) Absorbance changes upon photoswitching in IrisFP. The absorption spectrum of the on state (green) is characteristic of the chromophore in an anionic state, whereas the absorption spectrum of the off state (gray) shows the chromophore in a neutral state. Emission and excitation spectra are shown in dotted and dashed lines, respectively. Arrows highlight spectral evolution during switching; (**c**,**d**) Fluorescence emission by an IrisFP crystal in the green (on) and off states; (**e**,**f**) Electron density maps of the chromophore pocket of IrisFP in the on and off states (upon cyan light illumination, cyan lighting), overlaid on refined models of the protein in the two states. Chromophore isomerization and pocket reorganization are clearly visible; (**g**,**h**) Scheme of the reaction: isomerization of the chromophore (curved arrows) results in protonation of the phenolate moiety, resulting in a reversible nonfluorescent state.

Upon illumination of native crystalline IrisFP with 488 nm light, the crystals lose their color and become nonfluorescent. This nonfluorescent state is thermally metastable (t_1/2_ = 5.5 h), giving ample time to flash cool the crystals using the trigger freeze approach. Following illumination with 405 nm light at room temperature, the samples recover their yellow color and fluoresce again. This photocycle can be repeated many times with only minor decay of the fluorescence level in the on state due to irreversible photobleaching (Figure 5a). The crystallographic data clearly reveal *cis*-*trans* isomerization of the chromophore as a major determinant of photoswitching (Figure 5e–h). This is not very surprising after all, as the free p-HBI chromophore has a natural tendency to twist and isomerize upon photoexcitation instead of emitting fluorescence. The major role of the β-barrel in fluorescent proteins is thus to restrict this natural tendency as much as possible, i.e., rigidifying the chromophore, so that motionless radiative de-excitation pathways are promoted. When the chromophore pocket becomes less tight (due to the F173S mutation in IrisFP), isomerization becomes a likely pathway again. In contrast to photoconversion, photoswitching in IrisFP is associated with substantial structural rearrangements of the chromophore pocket, resulting in profound reorganization of hydrogen-bonding networks. The tightly H-bonded triad Glu144-His193-Glu211 in the cis configuration is replaced by the Glu144-Arg66-Glu211 triad in the trans configuration, with either His193 or Arg66 stabilizing the chromophore by π-stacking and π-cation interactions, respectively. Importantly, the trans configuration of the chromophore per se is not sufficient to explain the lack of fluorescence in the off state of IrisFP. Indeed, there exist fluorescent proteins with a trans chromophore that are strongly fluorescent. The major reason for the loss of fluorescence upon switching stems from protonation of the chromophore, as revealed by UV-Vis absorption data (Figure 5b). Therefore, cis-trans isomerization places the chromophore in a different local environment that strongly increases its pKa, so that, at physiological pH, the nonfluorescent protonated state prevails. This finding highlights the importance of combining kinetic crystallography with optical spectroscopic data to derive mechanistic insight into PTFP photophysics. Other reversibly switchable fluorescent proteins (RSFPs) of Anthozoan origin such as Dronpa [38] or mTFP0.7 [39] exhibit photoswitching mechanisms nearly identical to that of IrisFP. Recently, discovered RSFPs from Hydrozoan origin such as rsEGFP2 or rsFolder [15], or rsGreen [17] also display similar photoswitching mechanisms based on chromophore isomerization/protonation, although the chromophore pocket rearrangements differ.

Once in its photoconverted red state, IrisFP is still able to photoswitch. However, a clear structural view of the associated nonfluorescent state is difficult to obtain, as crystals must first be treated with 405 nm light to generate the red state and then with 561 nm light to induce off switching. Such harsh treatments are unfortunately prone to result in a limited occupancy of the targeted state, as well as promotion of a possible degradation of the crystalline order, producing suboptimal crystallographic data.

**Figure 6:**
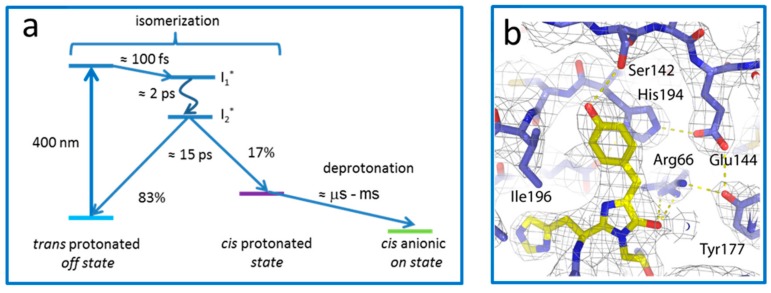
Investigation of off-on photoswitching of IrisFP by ultrafast methods. (**a**) Photoswitching pathway suggested by ultrafast absorbance spectroscopy; (**b**) View of the IrisFP chromophore pocket (green state) derived from serial crystallography data collected at the LCLS XFEL source. Figure prepared from refernece [24].

The exact photoswitching reaction pathway in RSFPs has fostered focused attention in recent years, notably to assess how acid–base chemistry couples with chromophore isomerization [7]. The question has been mostly addressed in the case of off to on (backward) photoswitching, a process that takes place with much higher quantum yield than (forward) on to off photoswitching and is thus easier to investigate experimentally. Starting with a protonated chromophore in the trans isomeric state, the hypothesis of an excited state proton transfer (ESPT) being the driving force for back photoswitching competes with that of isomerization taking place in the protonated state of the chromophore, followed by deprotonation in the cis conformation as a ground state process. Several investigations by ultrafast infrared and UV-Vis spectroscopy on Dronpa suggest that the latter hypothesis is the correct one [40,41]. In IrisFP, transient femtosecond absorption spectroscopy suggested the existence, upon excitation of the trans protonated state, of two excited-state intermediates with lifetimes in the picosecond regime, which may represent twisted chromophore species, possibly leading to isomerization (Figure 6a). A much longer-lived intermediate was also found and assigned to subsequent chromophore deprotonation [24], consistent with the results obtained on Dronpa. However, a direct proof by combined time-resolved spectroscopy and crystallography is yet to be obtained. The advent of XFEL sources could provide an excellent opportunity to reveal the near-atomic structure of such photoswitching intermediates. Preliminary experiments on IrisFP have recently demonstrated that microcrystals of this RSFP are of sufficient quality to solve the static structure of the green-emitting state by serial crystallography at the Linac Coherent Light Source (LCLS) (Figure 6b) [24], opening the door to ultrafast time-resolved structural studies on RSFPs.

**Figure 7:**
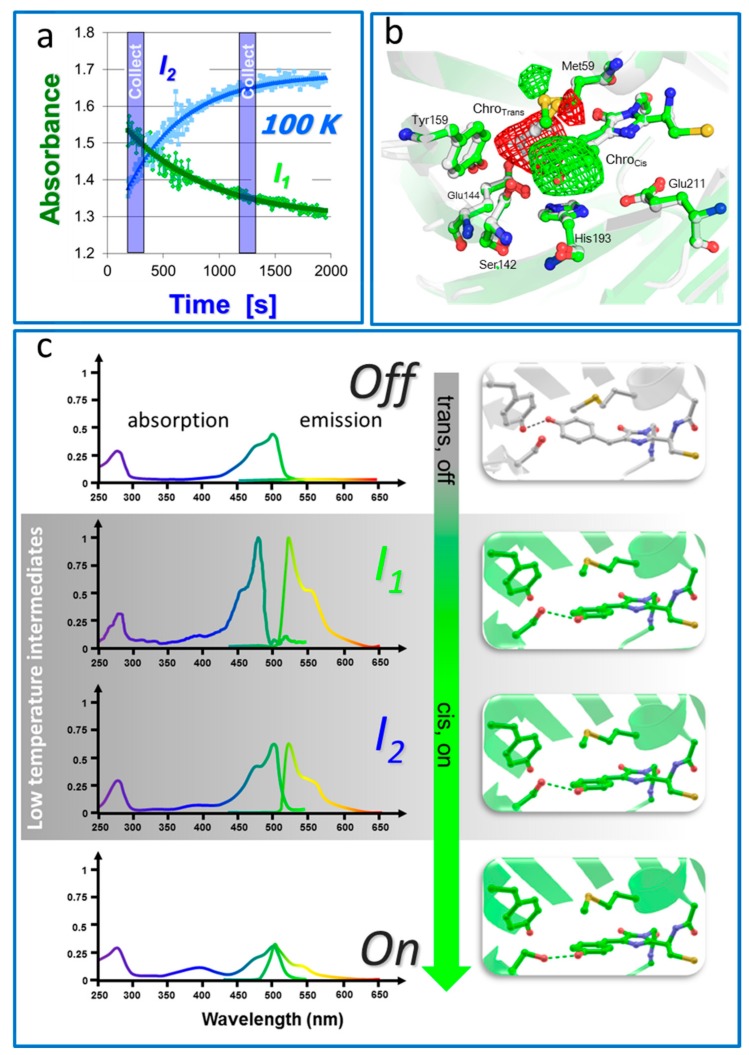
Investigation of off-on photoswitching of Padron by time-resolved low-temperature crystallography. (**a**) Time-evolution of two intermediates I1 and I2 building up upon illumination of Padron in its off state at cryogenic temperature; (**b**) Conformational change from the off state to the I1 state seen by cryocrystallography. The experimental difference electron density F_obs_–F_obs_ map (green: positive; red: negative) is overlaid on the models of Padron in its off (gray) and I1 (green) states; (**c**) Off to on reaction pathway of Padron. Absorption and emission spectra from the off, I1, I2 and on states, respectively, are shown on the left and corresponding structures are showed on the right. Figure 7C prepared from reference [2].

Another strategy to study photoswitching pathways in RSFPs consists of designing temperature (or pressure) dependent experiments to trap or prolong the lifetime of intermediate states of interest. Recent work on the RSFP Padron provides an interesting example [28]. Padron is a “positively switchable” RSFP, i.e., light used to excite the fluorescence of Padron also switches the protein on, instead of switching it off as in “negatively switchable” RSFPs such as Dronpa or IrisFP. By combining time-resolved cryo-X-ray crystallography at the Swiss Light Source (SLS), in crystallo spectroscopy, and molecular dynamics simulations, two fluorescent intermediates along the on-switching pathway of Padron were structurally and spectroscopically characterized (Figure 7). The data revealed unambiguously that, in Padron, *trans-cis* isomerization of the chromophore may occur purely in the anionic state and precedes protonation. The results are somewhat counterintuitive in terms of protein dynamics: they show that full *trans-cis* isomerization of the chromophore may take place in Padron at 100 K, a temperature at which dynamical breathing of proteins is essentially stalled. Such isomerization represents a very substantial structural modification, thus would not be expected at such a low temperature. It is possible that absorption of a visible photon provides sufficient thermal energy on the ultrafast timescale to allow the protein scaffold to transiently accommodate this substantial motion. A similar effect was recently observed in phytochrome [21]. Furthermore, it was observed in Padron that protonation of the chromophore following isomerization only takes place above the glass transition temperature T_g_, suggesting that protonation involves interactions with the solvent mediated by pronounced dynamical breathing of the protein scaffold. However, this protonation step did not involve any obvious structural modification of the protein, as judged from the crystallographic data. This study on Padron, beyond providing insight into the switching mechanism of this RSFP, challenges the common view that large structural changes (here, chromophore isomerization) in macromolecules may only take place at high temperatures in a highly flexible matrix, whereas small structural changes (here, chromophore protonation) are generally compatible with a reduced flexibility and may take place at low temperatures.

## 6. Photoblinking

Photoblinking is a stochastic process resulting in transient loss of fluorescence, thus somewhat resembles photoswitching. However, photoblinking can be differentiated from photoswitching by the fact that the dark states are generally produced with low quantum yields and are short-lived so that it is typically impossible to drive the entire population of an ensemble of fluorophores in a single blinked state at room temperature. Instead, only a minor fraction is typically found in the blinked state, which prohibits structural characterization due to insufficient occupancy in the crystal. However, deciphering blinking mechanisms in PTFPs is essential, as uncontrolled dark states represent major nuisances in advanced single-molecule based imaging applications, although the phenomenon can also be used to an advantage in some cases [42].

**Figure 8:**
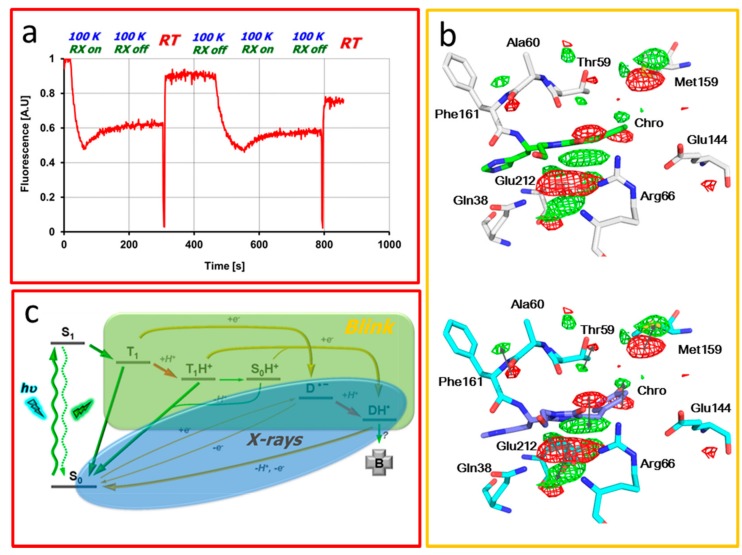
Photo blinking in IrisFP (**a**) Fluorescence profile of an IrisFP crystal recorded online upon repeated X-ray illumination (RX) at 100 K followed by transient annealing to room temperature (RT); (**b**) Conformational change due to X-ray induced blinking seen by cryocrystallography. The experimental difference electron density FO–FO map (green: positive; red: negative) is overlaid on the models of IrisFP in its on (gray, chromophore in green) and blinked (cyan, chromophore in blue) states. Arrows indicate the direction of motions; (**c**) Photophysical Jablonski scheme highlighting how X-ray illumination populates a radical blinked state via a shortcut pathway.

The possibility of structurally investigating blinking in IrisFP was discovered serendipitously [27]. When IrisFP crystals maintained at cryogenic temperature (100 K) are submitted to an X-ray beam, they exhibit a rapid loss of fluorescence emission, as demonstrated by in cristallo spectroscopic monitoring (Figure 8a). Interestingly, if the X-ray beam is blocked, a progressive and limited recovery of fluorescence emission is observed, suggesting that the photophysical mechanism at play is at least partially reversible. If the crystal is transiently annealed to room temperature, fluorescence almost completely recovers. The cycle can be repeated several times, suggesting the superposition of a major reversible process and a minor nonreversible one that can be associated with photobleaching. As *cis-trans* isomerization of the IrisFP chromophore does not take place at cryogenic temperature (contrary to the case of Padron), the reversible loss of fluorescence differs from photoswitching. Furthermore, because the process is induced by X-rays, it is likely that a red-ox mechanism is at play. Indeed, when they interact with solvent or with protein material, X-rays release free electrons (and electron holes) that generate strong reductive (or oxidizing) potential. We thus reasoned that the dark state produced in IrisFP crystals under these conditions could relate to blinking, as blinking mechanisms have often been reported to be redox driven in small organic fluorophores [43]. As most other photophysical pathways are blocked at cryogenic temperature, as the dark state is well stabilized at 100 K, the likelihood of a significant fraction of molecules being found in the dark state was high. Notably, the X-ray dose required to maximize dark-state formation in the crystals while minimizing nonreversible bleaching was low. Thus, the technique of composite diffraction data collection was used, in which small subsets of diffraction data are collected in different volumes of the needle shaped samples. A “very low-dose” data set was collected in this way, in which little dark state was predicted to form, compared to a “low-dose” data set, in which a substantial dark state was expected. Calculation of difference electron density maps between the very low and low dose data sets revealed conformational changes between the emissive and dark state structures of IrisFP (Figure 8b). The main observed feature is a clear distortion of the chromophore, whose methylene bridge seems to have lost the sp2 hybridization character of its central *Cα* carbon atom, consistent with a transient loss of π-conjugation and fluorescence. Chromophore distortion is associated with subtle motions of a limited number of residues in the chromophore pocket that can be accommodated at cryo-temperature within the rigidified protein matrix.

Clearly, the dark state observed in this experiment was not produced following absorption of a visible photon and therefore its photophysical relevance has to be questioned. A plausible hypothesis, however, is that the X-rays provided a shortcut to reach a genuine blinked state due to the very strong reductive power resulting from X-ray induced water radiolysis (Figure 8c). This state would normally be reached upon visible photon absorption to the excited state S1 followed by intersystem crossing to the triplet state T1 from which red ox chemistry ending up in a blinked state may occur. To evaluate such a scenario, a hybrid QM/MM approach was taken in conjunction with reaction-path finding methods and molecular dynamics simulations [44]. The simulations indeed suggested that a distorted chromophore displaying a structural signature highly similar to that observed experimentally could be generated upon electron transfer in the electronically excited state T1, producing a radical state with ruptured π-conjugation. However, it was found that the distortion also necessitated a coupled proton transfer to the chromophore atom *Cα*. Arginine 66, despite its normally high pKa, was identified as the most likely candidate for transient proton donation. Altogether, the complementary X-ray, spectroscopic and theoretical data provided a consistent picture of how blinking may occur in PTFPs of Anthozoan origin similar to IrisFP.

However, single-molecule imaging experiments demonstrated the co-existence of long-lived and short lived blinked states in mEos2, a monomeric variant of EosFP that is structurally very similar to IrisFP. The careful study of the photophysical properties of these states suggested that the distorted chromophore identified by crystallography corresponds to a short-lived blinked state, whereas a longer-lived state would exhibit a protonated chromophore at the phenolic oxygen with a yet unknown structure [11].

It is unlikely that such results on blinking could have been obtained by room temperature time-resolved crystallography. This is due to the multiple pathways that can be taken upon illumination at RT, each with rather low quantum yield. This kind of situation thus requires indirect tricks to be used. Here, low-temperature provided substantial advantage, as most pathways were arrested, redirecting photophysics towards a much reduced number of accessible states that then display tractable occupancies. Nevertheless, the possibility of generating artifactual results must always be kept in mind when such strategies are used, so that theoretical investigations are crucial to back up the experimental data.

## 7. Photobleaching

Capturing irreversibly photobleached states in fluorescent protein’s crystals poses a different challenge. Here, the small photobleaching quantum yield (typically of the order of ~10^−5^) is not so much an issue, since bleached molecules progressively accumulate as illumination is prolonged and thus rapidly form a relatively significant fraction of the crystal content. Therefore, a room temperature approach can be envisaged; crystals can in principle be illuminated by visible light in the same conditions as in real microscopy experiments. However, a different problem arises—that of crystal damage induced by excess light. Indeed, prolonged illumination or strong laser power densities imposed on crystalline samples may quickly result in cell expansion and loss of high-resolution diffraction, hindering the possibility of obtaining high quality difference electron density maps.

Nevertheless, the strong susceptibility of fluorescent proteins to photobleaching continues to be a major concern in the microscopy field. The generally rapid photodegradation of FPs is somewhat paradoxical, as, contrary to the case of organic dyes, the chromophore in FPs is relatively well protected from the solvent and notably from oxygen. Additionally, photobleaching rates are typically strongly dependent on experimental conditions, varying as a function of illumination mode (widefield or confocal), power and wavelength [45,46]. Thus, deciphering structural modifications associated with photobleaching in fluorescent proteins is an important step towards the rational engineering of photoresistant variants.

**Figure 9:**
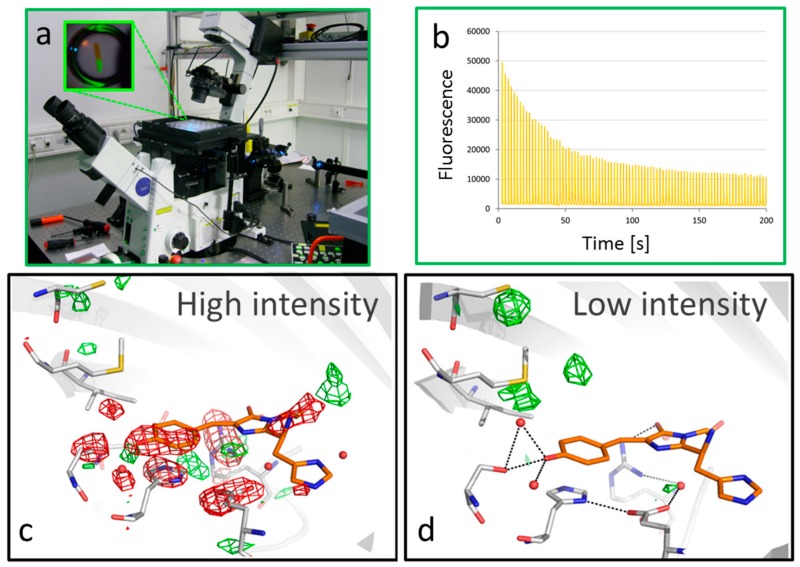

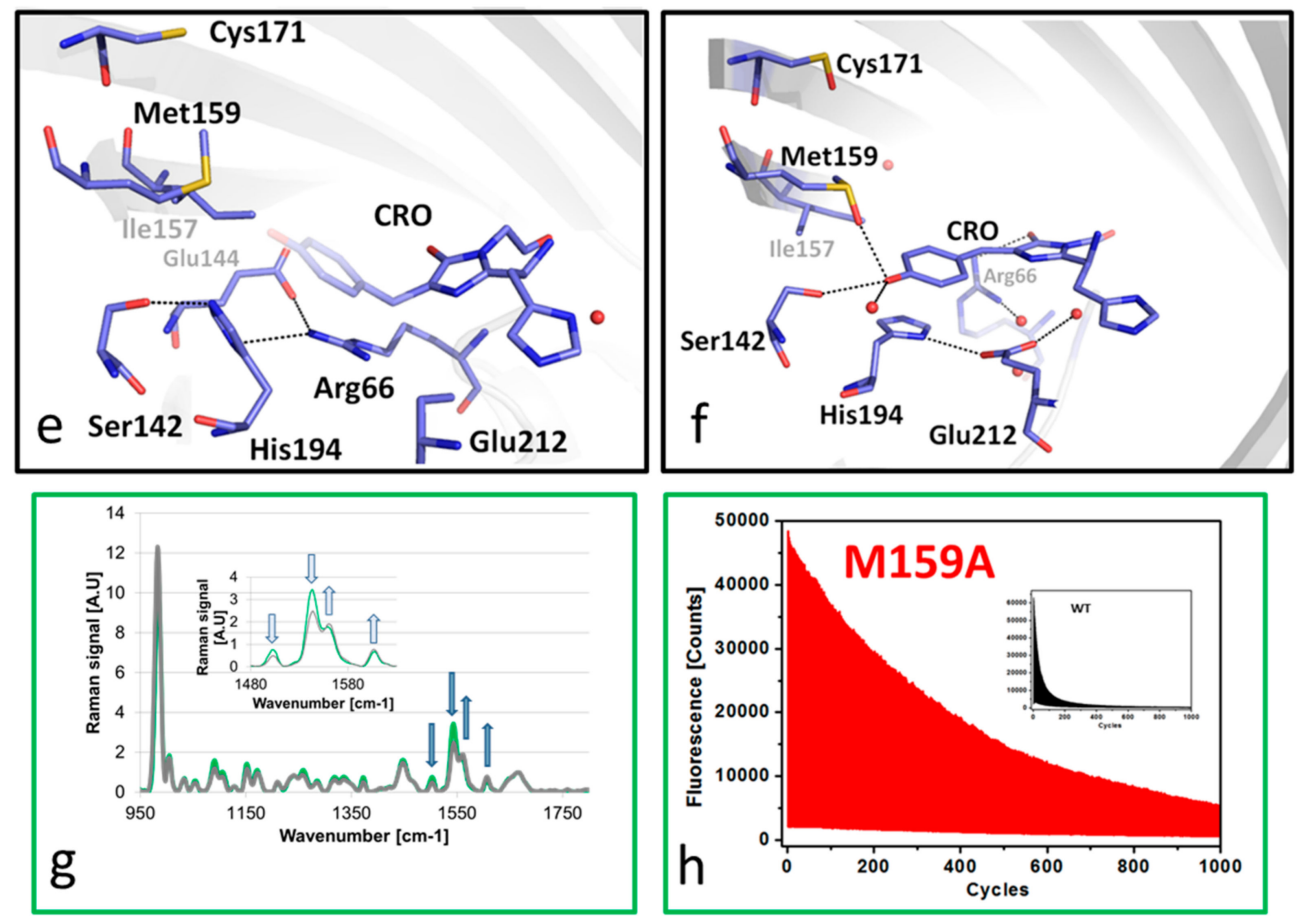
Photobleaching in IrisFP (**a**) PALM microscopy-based setup used to induce photobleaching in IrisFP crystals. The inset shows a crystal illuminated on half of its length; (**b**) Photofatigue of an IrisFP crystal. The plot shows repeated photocycles with progressive decay of the fluorescence signal due to irreversible photobleaching; (**c**,**d**) Difference electron density maps obtained upon photobleaching at high (low) illumination intensity (red: negative; green: positive) overlaid on the model of green IrisFP; (**e**,**f**) Refined models of the chromophore pocket in the photobleached states under high- (**e**) and low-intensity (**f**) illumination; (**g**) Raman signature of low intensity photobleaching (green: before illumination; gray: after illumination). The arrows point at changes characteristic of chromophore phenolate protonation (zoomed in the inset); (**h**) Photofatigue decay of the photoresistant variant IrisFP-M159A, under low intensity illumination. The inset shows the equivalent decay of the native protein.

To study photobleaching in IrisFP, we exposed crystals to repeated photoswitching using a homemade single-molecule based super-resolution fluorescence (PALM) microscope (Figure 9a) [13]. This allowed use of experimental conditions near those employed in nanoscopic approaches, where PTFPs are mostly used. Exposing the crystal to alternate 488 and 405 nm light allowed repeated switching between the on and off states of the IrisFP chromophores, progressively inducing a photobleached state through “photofatigue”, as can be seen by monitoring the decay of fluorescence emission during the process (Figure 9b). Once photobleached on approximately half of their volume, the needle shaped crystals were then flash cooled; diffraction data collected on both the intact and damaged parts so as to compute experimental difference density maps. Most of the time, the procedure failed: if too much photo fatigue was induced, then diffraction quality was lost; if limited photo fatigue was applied, the generated signal was typically swamped into noise due to laser induced cell expansion that considerably degraded the quality of the difference maps. It turned out that, in only one case, high-quality data could be recorded, showing the considerable extent of structural modifications induced by the high power-density laser light typically used in super-resolution microscopy (0.1 kW/cm²) (Figure 9c). The main observation is the decarboxylation of the conserved Glu212, likely associated with electron transfer to the chromophore through a Kolbe mechanism; the collapse of the entire H-bond network that normally maintains the chromophore rigidly flat. The irreversible loss of the π-conjugation of the chromophore can be unambiguously confirmed by Raman spectroscopy on crystals treated in exactly the same way. Indeed, the normally strong Raman band at 1565 cm^−1^ associated with the conjugated chromophore completely vanishes in the photobleached crystals. The photobleaching mechanism evidenced in these experiments was also found to be oxygen independent—to build up in a quadratic manner as laser power was increased. In total, a redox process dependent on the subsequent absorption of two photons was invoked; complementary molecular dynamics simulations provided a putative chemical structure for the damaged chromophore [13].

The great difficulty in obtaining well diffracting photobleached IrisFP crystals prompted us to strongly reduce the illumination power used to photofatigue the samples. Experiments were thus repeated with a 10-fold lower power density, i.e., 10 W/cm², more typical of a standard widefield microscopy experiment. The same overall pattern of photo fatigue decay was observed, albeit on a longer time scale. However, unexpectedly, striking differences were observed in the electron density maps when compared to those obtained under high-intensity illumination (Figure 9d). The chromophore appeared intact while positive features flanked the nearby Met159 and Cys171 residues, suggestive of sulfoxidation reactions. Chromatography coupled to electron spray ionization (ESI)-trap mass spectrometry on dissolved crystals photobleached in the same manner confirmed the presence of sulfoxidized Met159 and Cys171, as well as other oxidized residues located further from the chromophore pocket. These results are strongly indicative of an oxygen-dependent photodegradation pathway likely to involve singlet oxygen. It was proposed that under low illumination intensity not only was the photon absorption rate by IrisFP insufficient for promotion of the two-photon-based redox photobleaching mechanism described above, but also that the diffusion of molecular oxygen into the chromophore pocket was sufficient to keep up with that absorption rate. Thus, the chromophore, upon intersystem crossing to the triplet state T1, was able to react with molecular oxygen sitting within the chromophore pocket so as to produce singlet oxygen, which then reacted rapidly with the nearby sulfur containing residues. Now, as the chromophore was apparently left intact, why is it that the protein was irreversibly photobleached under these conditions? The answer came from Raman spectroscopy (Figure 9e) as well as UV-Vis absorption spectroscopy [13], which both revealed that the chromophore was trapped in a protonated, i.e., nonfluorescent, state, displaying a pKa of more than 11.5, whereas the normal pKa of the wild type IrisFP chromophore is around 6.0. “Trapped protonation” of the chromophore actually resulted from the presence of the strongly negatively charged sulfoxide moiety standing at hydrogen bond distance from the chromophore phenolate (Figure 9d). We have here an interesting example of a self Chromophore Assisted Light Inactivation (CALI) process, in which the IrisFP chromophore commits suicide by producing its own fatal weapon [13].

Another interesting observation is that the crystals photofatigued under these low intensity illumination conditions actually turned out to be even more degraded than when high-intensity illumination was used. This was attributed to the general oxidation of the protein, rapidly developing after the initial sulfoxidation of the two proximal residues Met159 and Cys171. Again here, only crystals in which a low-level of photobleaching was achieved could produce usable diffraction or Raman data, highlighting the delicate balance to be achieved between occupancy and crystalline quality when such experiments are attempted.

So far, improvement of photostability in FPs has mainly proceeded empirically using e.g., directed evolution strategies [45]. The structural view obtained for IrisFP immediately suggested mutation of Met159 into a non-sulfur containing residue. Although this mutation increased significantly the switching rate of the protein and somewhat reduced its brightness, it was found that its intrinsic photo resistance was indeed doubled under low illumination intensity (Figure 9f) [13].

## 8. Conclusions

The example of IrisFP demonstrates the potential of kinetic crystallography to elucidate the complex photophysical behaviours of fluorescent proteins, especially when used in combination with other complementary approaches that include optical spectroscopy, mass spectrometry, single-molecule imaging and theoretical calculations. Nevertheless, the multiple reaction pathways that fluorescent proteins may follow upon photon absorption, each with relatively low probability and each being potentially strongly influenced by experimental conditions, renders the comprehensive structural mapping of FP photophysics an extraordinary difficult task. Beyond our still limited understanding of light induced protein dynamics in general, it is clear that the prospect of engineering enhanced fluorescent proteins in a purely rational manner based on structural insight is still far ahead. As a matter of fact, the vast majority of popular fluorescent proteins used to date in cell biology labs have been obtained primarily by directed evolution approaches. A long route stands in front of us to bridge structural data, even recorded at the highest spatiotemporal resolution, with the ability to predict the effect of mutations in FPs. Particularly difficult are the combined effects of multiple mutations that typically display epistatic behaviour [47]. On the other hand, random approaches are not viable strategies when it comes to engineering subtle photophysical properties such as photoblinking in PTFPs, simply because screening millions of clones at the single-molecule level cannot be achieved with present technology. A compromise would be to attempt rational engineering of such properties based on e.g., kinetic crystallography data, combined with further rounds of random mutagenesis to overcome the generally unavoidable negative side effects (such as loss of brightness or shift in emission color) generated upon site directed mutagenesis. Thus, continued developments in the investigation of fluorescent protein’s structural dynamics will be crucial, both by emerging methodologies such as ultrafast serial crystallography at XFEL sources by more classical approaches based on intermediate state trapping, and monochromatic X-ray data collection at synchrotron sources. Even in IrisFP, with 12 structures already published in the protein databank, more fluorescent and nonfluorescent states still remain obscure and will need to be structurally deciphered in the near future.

## Abbreviations

FPs	Fluorescent proteins
GFP	Green fluorescent protein
PTFPs	Photo-transformable fluorescent proteins
PCFPs	Photo-convertible fluorescent proteins
RSFPs	Reversibly switchable fluorescent proteins
PALM	Photo activated localization microscopy
RESOLFT	Reversible saturable optical fluorescence transitions
XFEL	X-ray free electron laser
LCLS	Linac coherent light source
ESRF	European Synchrotron Radiation Facility
SLS	Swiss Light Source
ESI	Electron spray ionization
